# Paravalvular Leak

**DOI:** 10.14797/mdcvj.1096

**Published:** 2022-04-01

**Authors:** Fatima Qamar, Rowa H. Attar, Faisal Nabi

**Affiliations:** 1 Houston Methodist DeBakey Heart & Vascular Center, Houston, Texas, US

**Keywords:** paravalvular leak, prosthetic valve disease, mitral annulus

## Abstract

Despite improvements and advancements in surgical technique, paravalvular leaks (PVL) continue to present a challenge when caring for patients with prosthetic valve disease.^1^ Paravalvular leaks result from dehiscence of the surgical ring from the mitral annulus. Some theories suggest that uneven distribution of collagen fibers in the mitral annulus leaves the posterior mitral annulus without a well-formed fibrous structure, which may predispose it to recurrent mechanical injury that leads to PVL. The reported incidence of PVL is 2.2%.^2^ Risk factors associated with PVL include the presence of mitral annular calcification, infective endocarditis, active steroid use, and continuous surgical suturing, which poses a greater risk than an interrupted surgical approach.^3^ Risk of PVL varies by prosthesis type, with mechanical prostheses carrying a higher risk of PVL than bioprosthetic valves.

Below are images of a 70-year-old male with severe mitral stenosis and pulmonary hypertension who had previously undergone mitral valve commissurotomy and subsequent mitral valve replacement with a bioprosthetic mitral valve. He presented to the hospital with pulmonary edema. Initial transthoracic echocardiogram showed depressed biventricular function with a dehiscence of the bioprosthetic mitral valve and a large eccentric posterior PVL, severe tricuspid regurgitation, and severe pulmonary hypertension. Transesophageal images in ***Figure 1*** illustrate a significant posterior PVL with dehiscence of the prosthetic valve from the mitral annulus. The patient underwent closure of the paravalvular leak with two 18-mm Amplatzer ventricular septal defect occluders (Abbott) with excellent results and trace residual mitral regurgitation post closure (***Figure 2A, 2B***).

**Figure 1:**
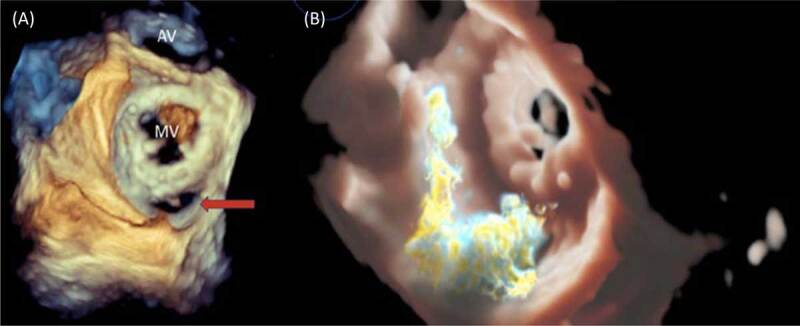
**(A)** 3-dimensional (3D) enface view of the mitral valve (MV) with the aortic valve (AV) seen in the 12 o’clock position. The mitral bioprosthesis is visualized with a paravalvular defect located posteromedially (red arrow). **(B)** 3D Trueview image of the mitral valve showing eccentric systolic flow through the paravalvular defect.

**Figure 2:**
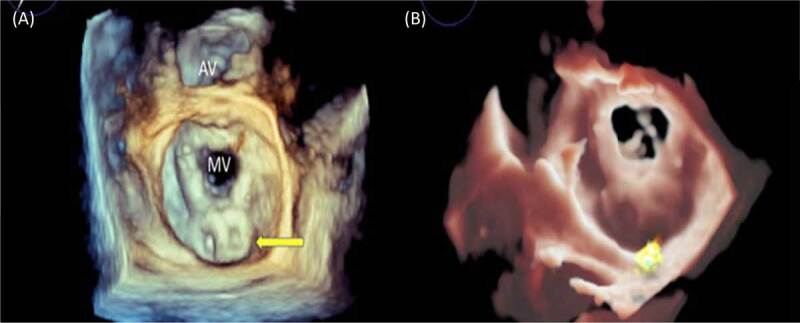
**(A, B)** A 3-dimensional enface view of the mitral valve with two Amplatzer ventricular septal defect (Abbott) devices (yellow arrow) well seated within the paravalvular defect. Panel B shows a small amount of residual paravalvular leak. AV: aortic valve; MV: mitral valve
